# Prevalence and Risk Factors of Lassa Seropositivity in Inhabitants of the Forest Region of Guinea: A Cross-Sectional Study

**DOI:** 10.1371/journal.pntd.0000548

**Published:** 2009-11-17

**Authors:** Solen Kernéis, Lamine Koivogui, N'Faly Magassouba, Kekoura Koulemou, Rosamund Lewis, Aristide Aplogan, Rebecca F. Grais, Philippe J. Guerin, Elisabeth Fichet-Calvet

**Affiliations:** 1 Epicentre, Paris, France; 2 Surveillance and Research Project on Viral Hemorrhagic Fevers (Lassa, Ebola and Yellow Fever) in West Africa, Conakry, Guinea; 3 Evolutionary Biology group, University of Antwerp, Antwerp, Belgium; Yale School of Public Health, United States of America

## Abstract

**Background:**

Lassa fever is a viral hemorrhagic fever endemic in West Africa. The reservoir host of the virus is a multimammate rat, *Mastomys natalensis*. Prevalence estimates of Lassa virus antibodies in humans vary greatly between studies, and the main modes of transmission of the virus from rodents to humans remain unclear. We aimed to (i) estimate the prevalence of Lassa virus–specific IgG antibodies (LV IgG) in the human population of a rural area of Guinea, and (ii) identify risk factors for positive LV IgG.

**Methods and Findings:**

A population-based cross-sectional study design was used. In April 2000, all individuals one year of age and older living in three prefectures located in the tropical secondary forest area of Guinea (Gueckedou, Lola and Yomou) were sampled using two-stage cluster sampling. For each individual identified by the sampling procedure and who agreed to participate, a standardized questionnaire was completed to collect data on personal exposure to potential risk factors for Lassa fever (mainly contact with rodents), and a blood sample was tested for LV IgG. A multiple logistic regression model was used to determine risk factors for positive LV IgG. A total of 1424 subjects were interviewed and 977 sera were tested. Prevalence of positive LV Ig was of 12.9% [10.8%–15.0%] and 10.0% [8.1%–11.9%] in rural and urban areas, respectively. Two risk factors of positive LV IgG were identified: to have, in the past twelve months, undergone an injection (odds ratio [OR] = 1.8 [1.1–3.1]), or lived with someone displaying a haemorrhage (OR = 1.7 [1.1–2.9]). No factors related to contacts with rats and/or mice remained statistically significant in the multivariate analysis.

**Conclusions:**

Our study underlines the potential importance of person-to-person transmission of Lassa fever, via close contact in the same household or nosocomial exposure.

## Author Summary

Lassa fever is a viral haemorrhagic fever endemic in West Africa, mainly transmitted to humans by multimammate rats. Several modes of virus transmission are suspected: aerosolisation of the virus, contact with infected rodent excreta, and consumption of rodent meat. Person-to-person transmission also occurs via contact with body fluids of infected persons (blood, urine) and is responsible for numerous outbreaks, mostly in healthcare facilities. Our objective was to precisely describe risk factors for Lassa fever in both rural and urban communities of forest Guinea. For each participant, a standardized questionnaire was completed and a blood sample tested for Lassa virus antibodies. A total of 1424 subjects were interviewed and 977 blood samples tested. The prevalence of Lassa virus antibodies was estimated at 12.9% and 10.0% in rural and urban areas, respectively. The two risk factors were: to have, in the past twelve months, undergone an injection, or lived with someone displaying a haemorrhage. Contrary to our expectation, no factors related to contact with rodents were identified. It is still probable that transmission occurs via indirect contact between rodents and humans in households, but our results highlight the importance of person-to-person transmission via close contact and nosocomial exposure.

## Introduction

Lassa fever (LF) is a viral haemorrhagic fever endemic in West Africa, caused by a single stranded RNA virus, member of the family *Arenaviridae* [1]. The disease is estimated to affect 2 million persons per year and cause 5,000 to 10,000 deaths [2]. It is expected to be largely under diagnosed as early symptoms are similar to those of other diseases such as malaria, leptospirosis or typhoid fever in endemic areas [3], and because many Lassa infections result in mild disease and never develop more specific symptoms associated with severe disease [2]. An adapted case definition for a suspected case has been defined, based on the observation of a large number of cases at Kenema Government Hospital in Sierra Leone (box S1) [4].

High rates of seroprevalence of Lassa fever have been reported in West Africa particularly in Guinea, Sierra Leone, Liberia and in Nigeria. In Cote d'Ivoire, Ghana, Togo and Benin, seroprevalence is lower, but isolated cases show evidence of viral circulation [5]–[7].

The reservoir host is the multimammate rat, *Mastomys natalensis*, first found infected in Sierra Leone and in Nigeria in 1972 [8],[9] and more recently in Guinea [10]. In northern Guinea, these commensal rodents aggregate in houses during the dry season, and disperse into gardens and the surrounding fields in the rainy season, foraging in cultivated areas before harvesting [11]. Several modes of virus transmission are suspected. Rodent-to-human infection could occur through the inhalation of aerosols or by direct contact with infected rodent excreta [12],[13]. Rodent consumption was also evoked as a possible risk behaviour [14],[15]. Person-to-person transmission occurs via direct contact with infected blood, urine, or pharyngeal secretions and is responsible for numerous outbreaks [16]–[22]. Most reported outbreaks have occurred in healthcare facilities in endemic areas, strongly suggesting a nosocomial transmission of the virus, probably through inadequate implementation of universal precautions, including reuse of syringes [23]. Nevertheless, Lassa serosurveys carried out in rural areas suggest that many villagers are infected in their communities. In Sierra Leone for example, communities studied between 1978 and 1983 had Lassa IgG seroprevalence ranging from 10% to 52% [13], and others in forest Guinea showed seroprevalence between 19% and 55% in 1991–1992 [24]. Sporadic cases are also documented among visitors to the region [6],[7],[25].

With the aim of identifying risk factors for transmission of Lassa virus, we performed a cross-sectional study in communities of the forest region of Guinea. In the same region, the team led by J. ter Meulen previously identified consumption of rodent meat as a potentially important risk factor for positive Lassa fever serology [14]. Therefore, we paid particular attention to exposures related to wild fauna, with the aim of further detailing which contacts with rodents (through collection, manipulation or consumption, etc.) would be at higher risk. Clinical signs during the twelve months before collection were also investigated to explore the association between symptoms compatible with Lassa fever and a positive Lassa serology.

In 1997, a research project on viral hemorrhagic fevers in the Mano River countries (Guinea, Liberia and Sierra Leone) and Ivory Coast was initiated by Ministries of Health, the University of Conakry, Epicentre, the Bernhard Nocht Institute in Hambourg (Germany), and the Institute Pasteur in Lyon (France).

## Methods

### Setting

**Figure 1:**
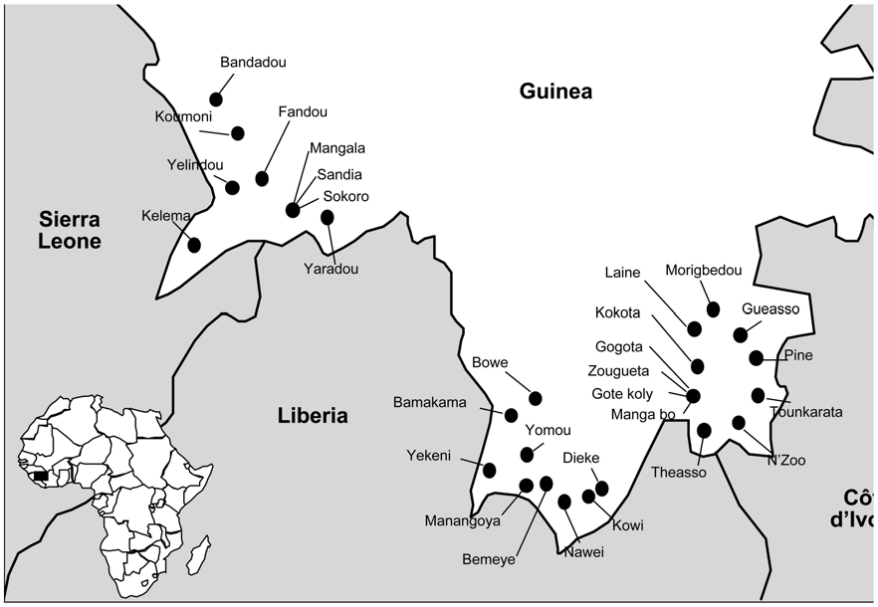
Location of 30 villages in Lassa fever serosurvey, Guinea, 2000.

We conducted a cross-sectional seroprevalence survey in three prefectures of Guinea, Guéckedou, Lola and Yomou, all located in the tropical secondary forest area of the country in April 2000 (figure 1). Two-stage cluster sampling was used for selection of villages/towns and households, following the standard WHO EPI survey method [26]. In the first step, villages or towns were selected with probability proportional to population size; secondly households; then finally one individual within each household. The list of towns and villages and population sizes for the sampling frame was provided by the regional health authorities, based on the most recent census (updated in the year 2000). Households were defined as persons living in the same dwelling, sharing meals and beds for at least the previous 6 months. In each selected village, the investigators started at a central point, randomly selected a direction from that point and chose one dwelling at random among those along the line from the centre to the edge of the village. Starting from this household, the next nearest household along the line was visited in turn until the size of the cluster was reached. Within each household sampled, one individual was randomly selected among individuals aged 1 year and older who had been living in the selected prefectures for more than 6 months.

### Data collection

Authorization for the survey had been obtained from the Guinea Ministry of Health and the University of Conakry. Both institutions approved oral consent prior to the initiation of the study, considering that the target population was predominantly illiterate. The purpose of the survey was explained to district administrative authorities, and then to village leaders and communities during public meetings a few days before investigation, and their agreement was requested. In each household, the investigators explained the survey objectives to the head of the household. Individuals identified by the sampling procedure were then invited to participate and informed oral consent was documented in the case report form for each participant. Consent was obtained by the investigators (nurses and/or physicians) in presence of the local guide, in order to improve adherence to the survey methodology and ensure appropriate translation of the questionnaire. Parents provided consent for children. The survey team consisted of six persons, working in pairs simultaneously in each prefecture and speaking the local dialect. Members of the survey teams underwent two days of training to familiarize themselves with the questionnaire and the sampling method.

The questionnaire was administered during an individual interview, including demographic data, questions on knowledge and beliefs on viral haemorrhagic fevers, information on personal exposure to potential risk factors, and clinical signs in the past twelve months. Specific questions concerned personal habits of collecting rodents, cutting up or eating dead rodents, professional occupation, contacts with other animals (monkeys, bats), history of surgical intervention or injection in the past twelve months, contact with a person displaying jaundice, haemorrhage, or someone who died of unknown causes. Our results derive from a study with Yellow fever as primary target disease. The project was later extended towards Ebola and Lassa fever, so that the questionnaire includes the exposure to animals which are not thought to harbour Lassa virus. Participants were asked about their current habits concerning contacts with rodents, and about injections and contact with people displaying hemorrhage etc. within the past twelve months.

After granting oral consent, participants provided a blood sample for Lassa virus–specific Immunoglobulin G (LV IgG) testing. Trained nurses collected all blood samples in accordance with a standardized procedure.

### Laboratory analysis

Specimens were analysed in the laboratory of the University hospital of Conakry. LV IgG were detected by indirect immunofluorescence assay using acetone-fixed Vero cells infected with Lassa virus strain Josiah according to the method described in ter Meulen et al. [14]. Immunofluorescence slides for Lassa fever serology were provided by the Bernhard Nocht Institute of Hamburg (Germany).

### Statistical analysis

Results are expressed as the median [min-max] for continuous variables and N (%) for categorical variables. The effects of continuous covariates were studied using Student's t-test or the Wilcoxon test when relevant. Fisher's exact tests were used for categorical variables.

A multiple logistic regression was performed to determine risk factors for positive LV IgG in our population. In the questionnaire, exposure to close contacts with rodents, bats, and monkeys was measured by a four-level ordinal variable (coded as ‘never’, ‘rarely’, ‘occasionally’ and ‘often’ exposed). This variable was transformed into a two-level categorical variable coded as: ‘never exposed’ versus ‘rarely, occasionally or often exposed’. In the region of ‘Guinée forestière’, we observed that the word ‘mouse’ usually refers to the domestic mouse or the multimammate mouse (*Mastomys*), contrary to the word ‘rat’ used to designate the black rat (*Rattus rattus*) or the giant rat (*Cricetomys gambianus*); the latter have never been reported as a reservoir for Lassa virus. Since it was impossible to confidently estimate the number of people who call mastomys a rat, we analysed exposure to rodents in two different ways: first by studying mice and rats separately, then by combining them into one exposure variable. All variables were included in the unadjusted analysis. We included in the multivariable model all variables achieving a P-value <0.25 in the unadjusted analysis, and all established risk factors for Lassa fever (mainly contacts with rodents), according to the published literature. The clustered structure of the data was taken into account in all tests by introducing a frailty term in the logistical regression models. We also adjusted for the prefecture term in all models. A backward stepwise variable selection procedure was applied to remove factors with P-value >0.05 (z-statistic).

Epi-Info was used to perform two-stage cluster sampling (Centers for Disease Control and Prevention [http://www.cdc.gov/epiinfo/]). Statistical analyses were carried out using R 2.4.0 statistical package (R Development Core Team; R Foundation for Statistical Computing, Vienna, Austria [http://www.R-project.org]).

## Results

Between 10 and 22 April 2000, in the 30 villages and towns selected, 1424 participants were interviewed and 1400 provided a blood specimen. Twenty-four participants did not give their consent for serological testing. Clusters were located in the prefectures of Gueckedou, Lola and Yomou (respectively 9, 12 and 9 clusters). Thirteen of the 30 clusters were considered to be ‘urban’ by the field investigators (defined as population size above 3,000 and easy road access).

Over the 1400 sera collected, 1347 were tested for Yellow Fever and only 977 for Lassa fever. Lassa fever immunofluorescence could not be conducted on all sera since the laboratory in Conakry did not have a sufficient supply of immunofluorescence slides to complete the Lassa serology tests. It was noted that sera from rural areas were less likely to be tested (proportions of non-tested sera were 36% and 26% respectively in rural and urban areas, p<10^−4^). No other differences (i.e. on age, gender) were observed between tested and non tested sera. Socio-demographic characteristics of all individuals included in the study are summarized in Table 1.

**Table 1:** Demographic characteristics of participants, Lassa fever serosurvey, Guinea, 2000.

	N (%)	N
	Median [IQR]	
Women	792 (56)	1424
Age (years)	30	1421
	[2–18–46–99]	
District		1424
Gueckedou	453 (32)	
Lola	514 (36)	
Yomou	457 (32)	
Occupation		1344
Housewife	564 (40)	
Farmer	330 (23)	
Apprentice/student	195 (14)	
Dayworkers/tradesman	75 (5)	
Shop owner	23 (2)	
Teacher	12 (1)	
Auxiliary midwife (“matrone”)	9 (1)	
Healthworkers	5 (1)	
No profession	94 (7)	
Others	37 (3)	
Religion		1397
Christian	584 (42)	
Muslim	395 (28)	
Animist	197 (14)	
No religion	202 (15)	
Other	19 (1)	
Positive Lassa virus–specific immunoglobulin G	112 (12)	977

Results are expressed as the median [min - 1st quartile - 3rd quartile - max] for continuous variables and N (%) for categorical variables.

**Figure 2:**
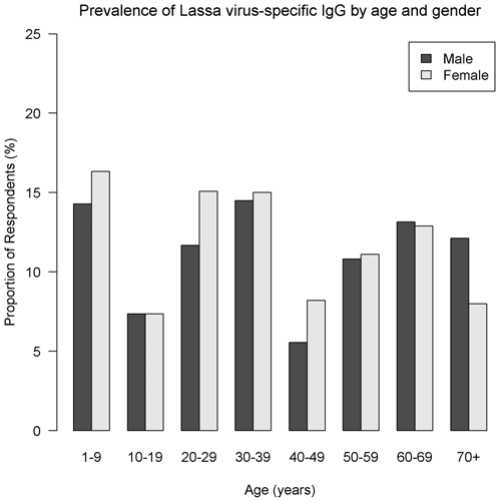
Prevalence of Lassa virus-specific IgG by age and sex, Guinea, 2000 (N = 975).

**Figure 3:**
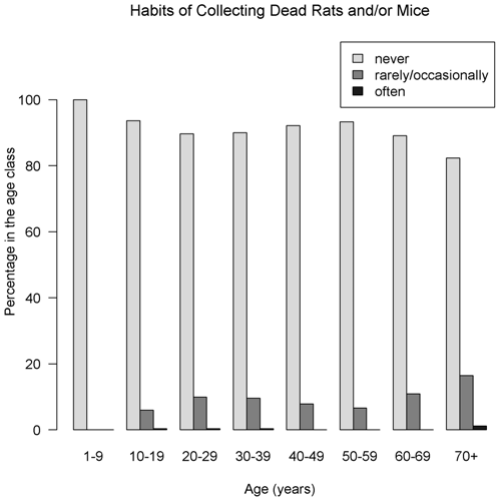
History of collecting rats and/or mice, Lassa fever serosurvey, Guinea, 2000 (N = 1404). Only a small proportion of subjects declared habits of collecting dead rats and/or mice in their everyday life. This practice seems to increase with age: for children under 10 years of age, no subject declared rare or occasional collection of rats and/or mice compared to 13% among those aged 70 and above.

**Figure 4:**
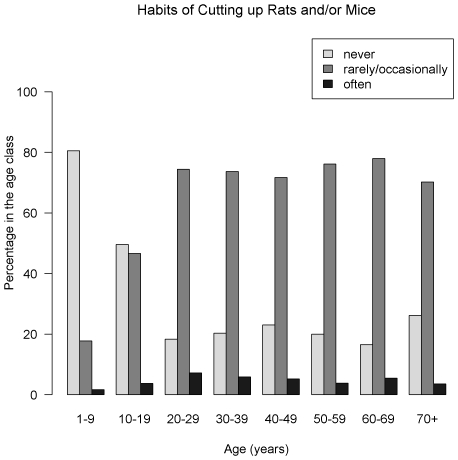
History of cutting up rats and/or mice, Lassa fever serosurvey, Guinea, 2000 (N = 1405). Habits of cutting up rats and/or mice were rare among children, and tended to increase with age to be relatively constant between age classes in adults.

**Figure 5:**
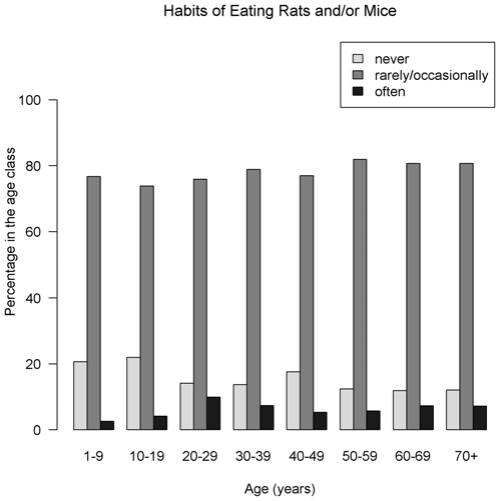
History of eating rats and/or mice, Lassa fever serosurvey, Guinea, 2000 (N = 1396). Habits of eating rats and/or mice were common among persons included in the study, and comparable in all age groups, 70–80% of persons declaring rare or occasional consumption.

The overall unadjusted prevalence of LV IgG was 11.5% (95% CI = [9.5–13.5]), and 11.3% [9.3–13.3] when adjusting for the variation in the proportion of samples tested between urban and rural areas. Seroprevalence was 12.9% [10.8–15.0] and 10.0% [8.1–11.9], in rural and urban clusters respectively. The seroprevalence by age and gender is summarized in figure 2. The most affected age-classes were: under 10 and between 20 and 29 years of age, in both sexes but differences were not significant (*P* = 0.54 and 0.66 respectively for females and males, Pearson's chi-square test). Figures 3, 4 and 5 show the distribution of the frequency of contacts with mice and/or rats according to age of the respondent (see tables S1, S2 and S3 for detailed numeric data). Only a small proportion of subjects reported collecting mice and/or rats in their everyday life. This practice seems to increase with age: no children under 10 years of age admitted collection of dead rodents, compared to 18% among those aged 70 and above (*P*<0.001, chi-squared test for trend). As shown in figure 4, a history of cutting up mice and/or rats was rare among children, and tended to increase with age (p<10^−4^, chi-squared test for trend). Figure 5 shows that eating mice and/or rats was common among persons included in the study, and comparable in all age groups, 70–80% of persons reporting rare or occasional consumption (figure S1 details data for each species).

Results of the univariate analysis of risk factors for positive LV IgG are shown in table 2. Variables achieving a P-value <0.25 in the marginal analysis were those concerning the location of the village (prefecture), type of residence (rural/urban), exposure to animals (work in a slaughterhouse, animal bites or scratches in the past twelve months), gathering of plants, use of traditional medicine, personal history of medical injection, or contact with someone displaying haemorrhage. Contacts with rodents (in particular mice and/or rats) were not significantly associated with positive LV IgG.

**Table 2:** Univariate analysis of risk factors for presence of Lassa virus–specific IgG.

Risk Factor	Lassa negative N = 865	Lassa positive N = 112	p	N
Prefecture†			0.003	977
Gueckedou	322 (37)	25 (22)		
Lola	289 (33)	52 (46)		
Yomou	254 (29)	35 (31)		
Age (years)†	30	30	0.73	975
	[4–18–45–90]	[5–20–41–89]		
Women	477 (55)	67 (60)	0.36	977
Rural area†	431 (50)	64 (57)	0.16	977
Activities (rarely, occasionally or often exposed to…)				
Collecting dead animals†				
Rats†	75 (9)	10 (9)	0.86	966
Mice†	78 (9)	11 (10)	0.73	966
Agoutis†	95 (11)	13 (12)	0.87	967
Squirrels†	76 (9)	13 (12)	0.38	967
Bats	81 (10)	13 (12)	0.49	967
Monkeys	74 (9)	13 (12)	0.29	959
Cutting up dead animals†				
Rats†	593 (69)	75 (68)	0.74	967
Mice†	570 (67)	72 (65)	0.74	966
Agoutis†	662 (77)	80 (72)	0.23	966
Squirrels†	674 (79)	81 (73)	0.18	966
Bats	541 (63)	73 (66)	0.68	964
Monkeys	598 (71)	73 (66)	0.32	959
Eating dead animals†				
Rats†	683 (80)	88 (80)	0.90	959
Mice†	665 (79)	82 (75)	0.33	956
Agoutis†	756 (89)	93 (85)	0.15	957
Squirrels†	685 (81)	84 (76)	0.25	957
Bats	648 (77)	84 (76)	0.81	949
Monkeys	686 (82)	84 (78)	0.29	944
Working in a slaughterhouse†	9 (1)	3 (3)	0.15	966
Caring of flock or herd	53 (6)	4 (4)	0.39	962
Gathering plants†	463 (54)	68 (61)	0.19	964
Taking traditional medicine†	652 (77)	96 (87)	0.01	962
Rats and/or mice in the household†	790 (93)	106 (96)	0.42	962
To have, in the last twelve months,				
Been bitten or scratched by an animal†	89 (11)	17 (15)	0.15	962
Undergone a surgical intervention	18 (2)	2 (2)	1	971
Had an injection†	546 (64)	87 (78)	0.004	967
Undergone a scarification	133 (16)	20 (18)	0.58	951
Lived in the same household as someone displaying jaundice	183 (24)	27 (27)	0.46	867
Lived in the same household as someone displaying hemorrhage† *	165 (21)	31 (32)	0.03	871
Lived in the same household as someone whodied of unknown causes	80 (10)	9 (9)	0.86	890
Participated in funeral rituals	109 (13)	16 (14)	0.65	960

Results are expressed as the median [min-max] for continuous variables and N (%) for categorical variables.

**†:** Included in the multivariable model (suspected risk factors and/or p<0.25).

*: Defined as: bleeding nose, bleeding gums, bloody diarrhea or blood coming from the eyes.

Both multivariable analyses (first with each rodent independently, and second with mice and rats taken together) gave the same result: no variable concerning exposure to rodents was significant in the final model. The two risk factors of positive LV Ig were to have, in the past twelve months: (i) undergone an injection (OR = 1.8 [1.1–3.1]); and (ii) lived with someone displaying a haemorrhage (OR = 1.7 [1.1–2.9]). The test on the frailty term was significant (p = 0.002), suggesting a heterogeneous cluster variation (design effect = 3.9).

Table 3 describes the clinical signs declared by the participants in the past twelve months. Signs that were significantly linked to positive LV IgG were those implying bleeding (bleeding nose, gums, bloody diarrhoea, or blood coming from the eyes) and jaundice. Hearing loss was not associated with presence of LV IgG; however, numbers of cases were small (respectively 19 and 3 in the LV IgG negative and positive groups). Other clinical signs, such as cough, sore throat, vomiting, did not seem to be specific of Lassa fever infection. It is important to note that 13% of participants with positive LV IgG reported no clinical signs in the past twelve months.

**Table 3:** History of symptoms among survey participants in the past twelve months by serologic status, Lassa fever serosurvey, Guinea, 2000.

Clinical signs	Lassa IgG negative N = 865	Lassa IgG positive N = 112	p	N
History of fever associated with bleeding nose, gums, or blood coming from the eyes	223 (26)	41 (37)	0.02	966
History of fever followed within fifteen days by jaundice and bleeding	115 (14)	23 (21)	0.04	962
Cough	286 (33)	44 (39)	0.24	970
Sore throat	205 (24)	32 (29)	0.29	966
Abdominal pain	514 (60)	77 (69)	0.08	972
Vomiting	151 (18)	25 (23)	0.19	965
Bloody diarrhea	274 (32)	54 (48)	0.001	970
Jaundice	115 (13)	24 (21)	0.03	969
Hearing loss	19 (2)	3 (3)	0.73	968
Bleeding nose or gums	114 (13)	23 (21)	0.04	966
Dermatological lesions	292 (34)	34 (31)	0.52	964
No clinical symptoms	111 (13)	14 (13)	1	969

## Discussion

Our study showed an adjusted overall prevalence of Lassa virus antibodies of 11.3% [9.3–13.3] in the year 2000, which is lower than previously published for the same region. In 1990–92, Lukashevich et al. estimated the seroprevalence for Lassa to be 23.6% (738/3,126) [24]. Lassa fever is a disease of rural areas influenced by the composition of the rodent population [10],[11]. Houses in villages close to roads, railways, commercial areas or with a higher human density harbour many domestic mice (*Mus musculus*) and black rats (*Rattus rattus*), but fewer *Mastomys* [27]. In suburban or peri-urban areas it is likely that the density of *Mastomys* and thus the risk for humans to contract the virus are lower. In our study, all villages of the region were eligible to be included with a probability proportional to population size, so that 13 of the 30 clusters (representing 46% of the study population) were located in urban areas (population size above 3,000 and easy road access), while Lukashevitch et al. collected samples in villages with 100–400 inhabitants only. This may explain why our estimation of the Lassa seroprevalence is lower than the one reported by Lukashevich et al. (32%, 447/1403 in Forest Guinea). Ter Meulen et al, who like us used the EPI sampling scheme in 1993, found a seroprevalence of 14.0% (105/751) [14], which is not very different from our result. Another possible explanation would be a lower sensibility of the Indirect Fluorescent Antibody test used in our study, compared to the technique used by Lukashevich et al. (ELISA with recombinant nucleocapsid protein antigen). And last, on the basis of data presented by Lukashevich et al. in 4 selected villages of the sample, the age structure of the population seems to differ from ours, with a much lower representation of children aged 5–14.

Another finding is that the prevalence of LV IgG does not appear to increase with age (figure 2), which was expected to be observed among people living in an endemic area of virus activity. This bimodal distribution is similar to those observed by Ter Meulen et al. in the same area, possibly reflecting different patterns of exposure, gathered from many localities with different epidemiological schemes [14]. This could also indicate that detectable Lassa virus IgG may not be long-lasting if exposure does not continue, as described for other viral hemorrhagic fevers [28].

In the multiple logistical regression model, two risk factors for positive serology were identified: having recently had an injection and having lived with someone displaying a haemorrhage. These two risk factors underline the importance of person-to-person transmission. Contrary to our expectation, we could not confirm that collection, manipulation or consumption of rodents were significant risk factors for positive LV IgG. Direct contact with rodents has always been suspected as a route of transmission to humans, based on several arguments: (1) the reservoir of the virus is a ubiquitous peridomestic rodent *Mastomys natalensis*; (2) rodents are infected in-utero and remain infective throughout life, excreting between 1000 and 10,000 infectious viral particles/mL of urine [2]; (3) there are geographical correlations between prevalence of LF in humans and rodents' infestation. Forest areas have a higher Lassa seroprevalence in humans and a higher occurrence of *Mastomys* [27] and houses with a higher proportion of infected rodents are associated with higher infection rates in human populations [2]. Consumption of rodents has been suspected as a possible route of disease transmission, but to our knowledge, this link has never been formally demonstrated. In a population-based study, Ter Meulen et al.have correlated possible risk factors for rodent-to-human transmission of Lassa virus with markers of Lassa Fever in two different regions of the Republic of Guinea. In one selected area, they found that 14.6% of rodent consumers had Lassa virus antibodies versus 7.4% of non-consumers, but the association failed to reach the conventional 5% significance threshold (*P* = 0.10) [14]. Despite in-depth questioning, habits of collecting, cutting up, or eating rodents was not associated with higher prevalence of LV Ig G, after adjusting for other factors. In our study, 46% of the study population lived in urban or peri-urban areas, and this modifies exposure to rodents. Misclassification due to the inclusion of people with contacts to agoutis, squirrels and rats in the exposed group may also explain why contact with rodents was not identified as risk factor for Lassa seropositivity. However, the hypothesis of transmission through rodent consumption is weakened by the observation that in rural areas, rodents are usually hunted far from houses in remote plantations (pers. obs.) whereas *M. natalensis*, the only reservoir of the virus, is mainly found around human dwellings [27].

Our study reinforces the importance of person-to-person transmission, within the same household or through nosocomial exposure, via contact with body fluids of ill persons. It has long been known that Lassa virus may persist at low levels in the urine of humans for up to two months after infection (without circulation in the blood) [29]. Even if there are no studies on the presence of Lassa virus in semen, sexual transmission during convalescence could be considered to be a possibility, like for other hemorrhagic fevers in Africa such as Ebola [30]. There is also anecdotal evidence suggesting venereal rodent-to-rodent transmission of the etiologic arenaviruses of three South American hemorrhagic fevers (Argentine, Bolivian and Venezuelan), which also emphasizes the possibility of sexual transmission of Lassa virus [31].

Our data on Lassa fever does not allow us to explore this hypothesis, since we have no information on the serostatus of the partners of the participants, and this could be an interesting topic for further research.

Several limitations can be noted. First, our results may have been affected by a recall bias over the 12 months recall period. However, since the results of LV Ig were unknown at the time of interview, such bias is unlikely to have affected the two groups (positive/negative serology) differently. Second, over the 1400 sera collected, only 977 were tested. Lack of reagents explains discrepancy between total samples and tested samples. Sera from rural areas were less likely to be tested (proportion of non-tested sera were 36% and 26% respectively in rural and urban areas, p<10^−4^), also suggesting transportation problems. This over-representation of habitants of urban areas may partly explain the relatively low seroprevalence of LV IgG observed in our study. Third, investigators may have had difficulties with the questionnaire, which was long and complex. Fourth, our sampling method slightly differs from the EPI cluster sampling as described by WHO. In our sample, only one eligible household member has been selected instead of all eligible household members, probably leading to an over-representation of individuals from small households. For a disease like Lassa fever with human-to-human transmission, it is likely that circulation of the virus is facilitated in large households compared with small ones. Consequently, people from small households are probably less likely to be infected, which may have led to a moderate underestimation of the prevalence. And finally, due the cross-sectional nature of the study and long recall period, it is not possible to establish a temporal relationship between risk exposure, clinical syndromes detected, and serology results. For example, we cannot ascertain whether participants with a history suggestive of Lassa fever had an injection before or after onset of symptoms, that is, whether the injection was a risk factor for or a consequence of Lassa fever. With our data, it was also impossible to distinguish between people who received an injection as a treatment of a possible Lassa fever, to those who have been vaccinated or treated for malaria.

Despite these limitations, our findings are consistent with previous observations of nosocomial transmission being an important factor for transmission of VHF, and particularly Lassa fever. Most documented Lassa Fever outbreaks have occurred in health care facilities in endemic areas, strongly suggesting inadequate implementation of universal precautions, e.g. reused syringes [23]. Limited resources may lead to sharing and reuse of disposable equipment, and increased risk of Lassa fever and other viral hemorrhagic fever outbreaks such as Ebola [23],[32]. Indications for parenteral treatment should be very restrictive in order to avoid unnecessary injections. This is difficult because people tend to believe that injections have superior therapeutic value [23]. In our study, 897 persons (64%) reported having received an injection in the past twelve months. This proportion is very high for a healthy group, sampled from the general population. No vaccination campaign had been conducted in the region prior to the study. It is likely that these injections were treatments against malaria, since in these rural regions, parenteral quinine was the treatment of choice in 2000.

In conclusion, our results did not confirm consumption of rodent meat to be an important risk factor for positive Lassa fever serology. However, it is still probable that transmission from rodents to humans occurs via indirect contact in households through exposure to food or fomites soiled with rodent excretions (faeces, urine etc) or to aerosolized virus. Close contacts with infected humans (in the same household or via nosocomial exposure) are also major routes of contamination. It is crucial to reinforce standard hygiene precautions, particularly in health care settings. Recent progress in development for a Lassa fever vaccine [33] offers new opportunities for prevention of this deadly disease. Evidence of nosocomial transmission of the disease suggests that health workers may benefit from such vaccine in the future.

## Supporting Information

Alternative Language Abstract S1 Translation of the abstract into French by SK.
(0.02 MB DOC)

Box S1 Case definition for a suspected case of Lassa fever (based on [4]).
(0.02 MB DOC)

Figure S1 Details on contacts with rodents and bats.
(0.09 MB TIF)

Table S1 Number of sera with positive Lassa virus-specific immunoglobulin G by village. Results are expressed as N (%).
(0.05 MB DOC)

Table S2 Serums with positive Lassa virus-specific IgG by age and sex. Results are expressed as N (%).
(0.03 MB DOC)

Table S3 Contacts with mice and/or rats by age. Results are expressed as N (% in the age class).
(0.04 MB DOC)

Table S4 Sample size for Figure S1.
(0.03 MB DOC)
