# Correction: Prevalence and Risk Factors of Lassa Seropositivity in Inhabitants of the Forest Region of Guinea: A Cross-Sectional Study

**DOI:** 10.1371/annotation/998b7c28-81b7-44f8-a76d-eb69e2d205d6

**Published:** 2010-01-22

**Authors:** Solen Kernéis, Lamine Koivogui, N'Faly Magassouba, Kekoura Koulemou, Rosamund Lewis, Aristide Aplogan, Rebecca F. Grais, Philippe J. Guerin, Elisabeth Fichet-Calvet

The first sentence in the Acknowledgments section should read: "We are indebted to Mohamed Sakho, Barré Soropogui, Amadou Doré and Fodé Kourouma for their contribution in the fieldwork."

