# Supplementary material for: Prevalence and Risk Factors of Lassa Seropositivity in Inhabitants of the Forest Region of Guinea: A Cross-Sectional Study
Source: PLoS Negl Trop Dis. 2009 Nov 17;3(11):e548. doi: 10.1371/journal.pntd.0000548 (PMC2771900; doi:10.1371/journal.pntd.0000548)
Supplement: Alternative Language Abstract S1 — Translation of the abstract into French by SK. (0.02 MB DOC) [file pntd.0000548.s001.doc]

**Translation of the abstract into French by S Kernéis**

*Contexte*: La fièvre de Lassa est une fièvre hémorragique virale endémique en Afrique de l’Ouest, dont le réservoir est un rongeur, le *Mastomys natalensis*. Les estimations de séroprévalence chez l’être humain donnent des résultats très variables d’une étude à l’autre et les modes de transmission du virus du rongeur à l’homme ne sont pas clairement définis. *Objectifs* : (i) Estimer la séroprévalence de la fièvre de Lassa (Ig G dirigées contre le virus de Lassa, IgG VL positives) dans une population de personnes vivant dans plusieurs régions rurales de Guinée Conakry, et (ii) Identifier les facteurs de risque de sérologie positive. *Méthodes / Principaux résultats*: Une enquête transversale par sondage en grappes à deux degrés a été menée en avril 2000 auprès des individus âgés de 1 an et plus vivant dans 3 préfectures (Gueckedou, Lola et Yomou) situées en Guinée Forestière. Pour chaque individu identifié par le tirage au sort, un questionnaire standardisé était rempli, collectant des informations sur les expositions à d’éventuels facteurs de risque de fièvre de Lassa (principalement les contacts avec les rongeurs), et un prélèvement sanguin était proposé pour réaliser un dosage des IgG VL. Une régression logistique a ensuite été utilisée pour identifier les facteurs de risque de sérologie positive. Au total 1424 participants ont été interrogés et 977 ont accepté le prélèvement sanguin. La prévalence des IgG VL positives était de 12,9% [10,8 – 15,0] et de 10,0% [8,1 – 11,9] respectivement dans les zones rurales et urbaines. Les deux facteurs de risque de sérologie Lassa positive étaient d’avoir, au cours des 12 derniers mois, reçu une injection (Odds Ratio, OR=1,8 [1,1 – 3,1]), ou vécu sous le même toit que quelqu’un ayant présenté des signes d’hémorragie (saignement nasal, oculaire, des gencives ou diarrhée sanglante), (OR 1,7 [1,1 – 2,9]). Les contacts avec les rongeurs n’étaient pas significativement associés à une sérologie positive dans l’analyse multivariée. *Conclusions* : Cette étude souligne l’importance de la transmission interhumaine du virus de Lassa, par contact étroit au sein du même foyer ou lors de soins médicaux.
