# Supplementary material for: Prevalence and Risk Factors of Lassa Seropositivity in Inhabitants of the Forest Region of Guinea: A Cross-Sectional Study
Source: PLoS Negl Trop Dis. 2009 Nov 17;3(11):e548. doi: 10.1371/journal.pntd.0000548 (PMC2771900; doi:10.1371/journal.pntd.0000548)
Supplement: Box S1 — Case definition for a suspected case of Lassa fever (based on [4]). (0.02 MB DOC) [file pntd.0000548.s002.doc]

Case definition for a suspected case of Lassa fever (based on [4]):

- Fever >38 °C for less than 3 weeks and,
- Absence of signs of local inflammation (i.e. the illness is systemic) and,
- Absence of a clinical response after 48 h of anti-malaria treatment and/or a broad-spectrum antibiotic and,
- Two major signs or one major sign and two minor signs described below:

**Major signs**

- Bleeding (including from the mouth, nose, rectum, or vagina)
- Swollen neck or face
- Conjunctivitis or subconjunctival hemorrhage
- Spontaneous abortion
- Petechial or hemorrhagic rash
- New onset of tinnitus or altered hearing
- Persistent hypotension
- Elevated liver transaminases, especially aspartate aminotransferase > alanine aminotransferase
- Known exposure to a person suspected to have Lassa fever

**Minor signs**

- Headache
- Sore throat
- Vomiting
- Diffuse abdominal pain/tenderness
- Chest/retrosternal pain
- Cough
- Diarrhea
- Generalized myalgia or arthralgia
- Profuse weakness
- Proteinuria
- Leucopenia <4000/L
