# Supplementary figures and images for: Prevalence and Risk Factors of Lassa Seropositivity in Inhabitants of the Forest Region of Guinea: A Cross-Sectional Study
Source: PLoS Negl Trop Dis. 2009 Nov 17;3(11):e548. doi: 10.1371/journal.pntd.0000548 (PMC2771900; doi:10.1371/journal.pntd.0000548)

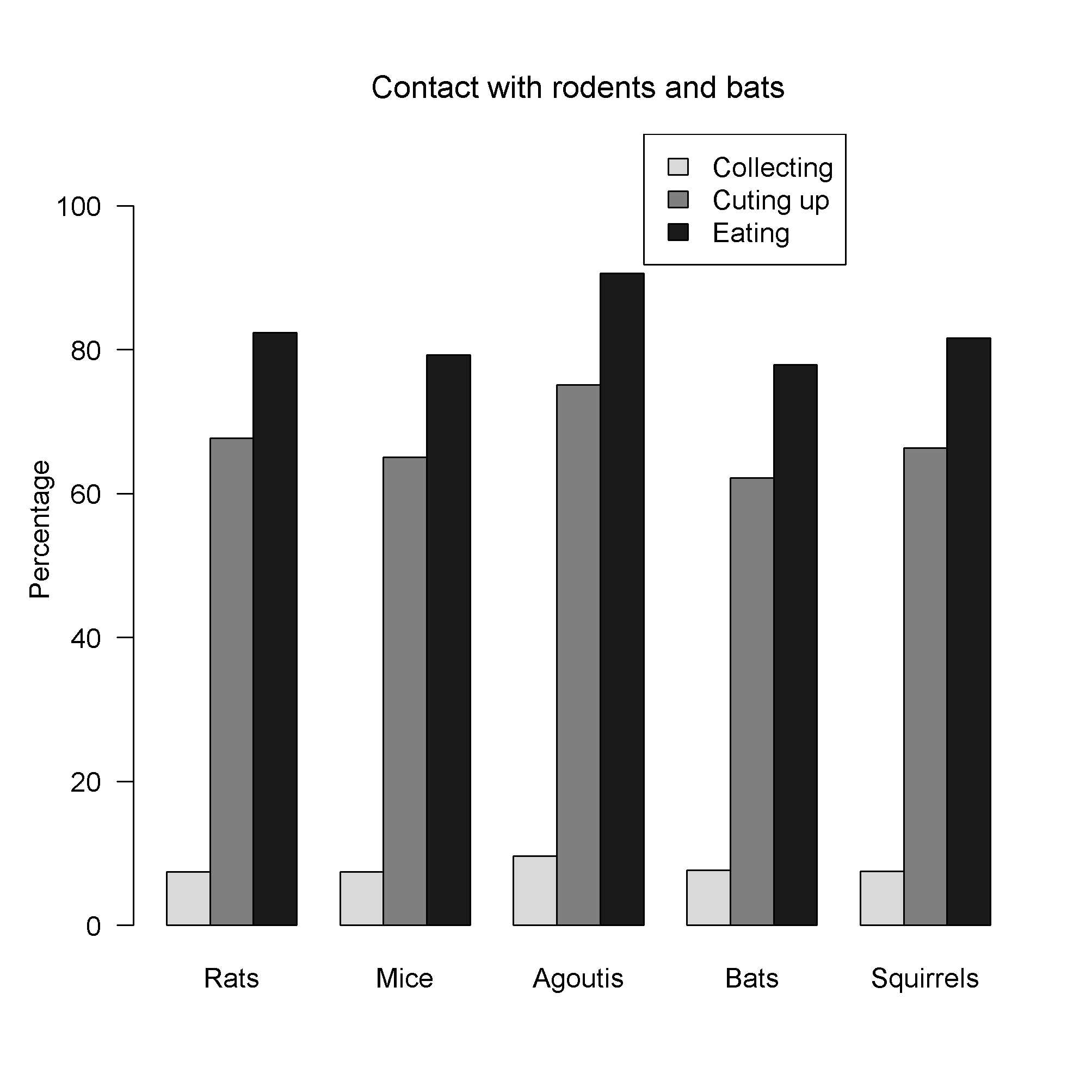

Supplement: Figure S1 — Details on contacts with rodents and bats. (0.09 MB TIF) [file pntd.0000548.s003.tif]
