# Supplementary material for: Prevalence and Risk Factors of Lassa Seropositivity in Inhabitants of the Forest Region of Guinea: A Cross-Sectional Study
Source: PLoS Negl Trop Dis. 2009 Nov 17;3(11):e548. doi: 10.1371/journal.pntd.0000548 (PMC2771900; doi:10.1371/journal.pntd.0000548)
Supplement: Table S1 — Number of sera with positive Lassa virus-specific immunoglobulin G by village. Results are expressed as N (%). (0.05 MB DOC) [file pntd.0000548.s004.doc]

Table S1: Number of sera with positive Lassa virus-specific immunoglobulin G by village. Results are expressed as N (%).

|  | Serum with positive Lassa virus–specific immunoglobulin G | Number of sera tested |
| --- | --- | --- |
| BAMAKAMA | 4 (15) | 26 |
| BANDADOU | 1 (2) | 41 |
| BEMEYE | 1 (11) | 9 |
| BOWE | 8 (27) | 30 |
| DIECKE | 3 (4) | 73 |
| FANDOU BENDOU | 5 (15) | 33 |
| GOGOTA | 0 (0) | 6 |
| GOTE KOLY | 7 (33) | 21 |
| GUEASSO | 1 (9) | 11 |
| KELEMA | 1 (4) | 23 |
| KOKOTA | 16 (31) | 51 |
| KOUMONI | 2 (5) | 40 |
| KOWI | 4 (14) | 29 |
| LAINE | 2 (5) | 37 |
| MANANGOYA | 0 (0) | 12 |
| MANGA BO | 0 (0) | 19 |
| MANGALA | 1 (4) | 26 |
| MORIGBEDOU | 4 (10) | 40 |
| NAWEI | 10 (19) | 53 |
| NZOO | 2 (6) | 35 |
| PINE | 15 (42) | 36 |
| SANDIA | 2 (4) | 51 |
| SOKORO | 2 (6) | 32 |
| THEASSO | 2 (8) | 25 |
| TOUNKARATA | 1 (2) | 47 |
| YARADOU | 7 (14) | 51 |
| YEKENI | 2 (25) | 8 |
| YELINDOU | 4 (8) | 50 |
| YOMOU | 3 (6) | 49 |
| ZOUGUETA | 2 (15) | 13 |
