# Supplementary material for: Prevalence and Risk Factors of Lassa Seropositivity in Inhabitants of the Forest Region of Guinea: A Cross-Sectional Study
Source: PLoS Negl Trop Dis. 2009 Nov 17;3(11):e548. doi: 10.1371/journal.pntd.0000548 (PMC2771900; doi:10.1371/journal.pntd.0000548)
Supplement: Table S2 — Serums with positive Lassa virus-specific IgG by age and sex. Results are expressed as N (%). (0.03 MB DOC) [file pntd.0000548.s005.doc]

Table S2: Serums with positive Lassa virus-specific IgG by age and sex. Results are expressed as N (%).

|  | Male | Female |
| --- | --- | --- |
| < 10 | 4 (14) | 8 (16) |
| 10 – 19 | 7 (7) | 7 (7) |
| 20 – 29 | 7 (12) | 19 (15) |
| 30 – 39 | 10 (14) | 18 (15) |
| 40 – 49 | 4 (6) | 5 (8) |
| 50 – 59 | 4 (11) | 4 (11) |
| 60 – 69 | 5 (13) | 4 (13) |
| ≥ 70 | 4 (12) | 2 (8) |
