# Supplementary material for: Prevalence and Risk Factors of Lassa Seropositivity in Inhabitants of the Forest Region of Guinea: A Cross-Sectional Study
Source: PLoS Negl Trop Dis. 2009 Nov 17;3(11):e548. doi: 10.1371/journal.pntd.0000548 (PMC2771900; doi:10.1371/journal.pntd.0000548)
Supplement: Table S3 — Contacts with mice and/or rats by age. Results are expressed as N (% in the age class). (0.04 MB DOC) [file pntd.0000548.s006.doc]

Table S3: Contacts with mice and/or rats by age. Results are expressed as N (% in the age class).

|  |  |  | Collecting |  |  |  | Cutting up |  |  |  | Eating |  |
| --- | --- | --- | --- | --- | --- | --- | --- | --- | --- | --- | --- | --- |
|  |  | Never | Rarely/  Occasionally | Often |  | Never | Rarely/  Occasionally | Often |  | Never | Rarely/  Occasionally | Often |
| < 10 |  | 116 (100) | 0 | 0 |  | 95 (80) | 21 (18) | 2 (2) |  | 24 (21) | 89 (77) | 3 (3) |
| 10 – 19 |  | 249 (94) | 16 (6) | 1 |  | 132 (50) | 124 (47) | 10 (4) |  | 58 (22) | 195 (74) | 11 (4) |
| 20 – 29 |  | 234 (90) | 26 (10) | 1 |  | 48 (18) | 195 (74) | 19 (7) |  | 37 (14) | 199 (76) | 26 (10) |
| 30 – 39 |  | 243 (90) | 26 (10) | 1 |  | 55 (20) | 199 (74) | 16 (6) |  | 37 (14) | 213 (76) | 20 (10) |
| 40 – 49 |  | 176 (92) | 15 (8) | 0 |  | 44 (23) | 137 (72) | 10 (5) |  | 33 (18) | 144 (77) | 10 (5) |
| 50 – 59 |  | 98 (93) | 7 (7) | 0 |  | 21 (20) | 80 (76) | 4 (4) |  | 13 (12) | 86 (82) | 6 (6) |
| 60 – 69 |  | 98 (89) | 12 (11) | 0 |  | 18 (17) | 85 (78) | 6 (5) |  | 13 (12) | 88 (81) | 8 (7) |
| ≥ 70 |  | 70 (82) | 14 (17) | 1 (1) |  | 22 (26) | 59 (70) | 3 (4) |  | 10 (12) | 67 (81) | 6 (7) |
