# Supplementary material for: Prevalence and Risk Factors of Lassa Seropositivity in Inhabitants of the Forest Region of Guinea: A Cross-Sectional Study
Source: PLoS Negl Trop Dis. 2009 Nov 17;3(11):e548. doi: 10.1371/journal.pntd.0000548 (PMC2771900; doi:10.1371/journal.pntd.0000548)
Supplement: Table S4 — Sample size for Figure S1. (0.03 MB DOC) [file pntd.0000548.s007.doc]

Table S4 : Sample size for figure S1.

|  | Collecting | Cutting up | Eating |
| --- | --- | --- | --- |
| Rats | 1406 | 1408 | 1399 |
| Mice | 1406 | 1406 | 1396 |
| Agoutis | 1406 | 1406 | 1396 |
| Bats | 1405 | 1401 | 1386 |
| Squirrels | 1405 | 1403 | 1392 |
